# 3D-printed PCL@BG scaffold integrated with SDF-1α-loaded hydrogel for enhancing local treatment of bone defects

**DOI:** 10.1186/s13036-023-00401-4

**Published:** 2024-01-02

**Authors:** Chenglong Wang, Jinlei Dong, Fanxiao Liu, Nan Liu, Lianxin Li

**Affiliations:** 1 Department of Orthopaedics Surgery, Shandong Provincial Hospital Affiliated to Shandong First Medical University, Jinan, 250021 China; 2 Department of Orthopaedics Surgery, Shandong Trauma Center, Jinan, 2500021 China

**Keywords:** Hydrogel, PCL, Bone defect, SDF-1α, 3D-printed scaffold, Bioactive glass

## Abstract

**Background:**

The long-term nonunion of bone defects is always a difficult problem in orthopaedics treatment. Artificial bone implants made of polymeric materials are expected to solve this problem due to their suitable degradation rate and good biocompatibility. However, the lack of mechanical strength, low osteogenic induction ability and poor hydrophilicity of these synthetic polymeric materials limit their large-scale clinical application.

**Results:**

In this study, we used bioactive glass (BG) (20%, W/W) and polycaprolactone (PCL, 80%, W/W) as raw materials to prepare a bone repair scaffold (PCL@BG20) using fused deposition modelling (FDM) three-dimensional (3D) printing technology. Subsequently, stromal cell-derived factor-1α (SDF-1α) chemokines were loaded into the PCL@BG20 scaffold pores with gelatine methacryloyl (GelMA) hydrogel. The experimental results showed that the prepared scaffold had a porous biomimetic structure mimicking that of cancellous bone, and the compressive strength (44.89 ± 3.45 MPa) of the scaffold was similar to that of cancellous bone. Transwell experiments showed that scaffolds loaded with SDF-1α could promote the recruitment of bone marrow stromal cells (BMSCs). In vivo data showed that treatment with scaffolds containing SDF-1α and BG (PCL@BG-GelMA/SDF-1α) had the best effect on bone defect repair compared to the other groups, with a large amount of new bone and mature collagen forming at the bone defect site. No significant organ toxicity or inflammatory reactions were observed in any of the experimental groups.

**Conclusions:**

The results show that this kind of scaffold containing BG and SDF-1α serves the dual functions of recruiting stem cell migration in vivo and promoting bone repair in situ. We envision that this scaffold may become a new strategy for the clinical treatment of bone defects.

**Supplementary Information:**

The online version contains supplementary material available at 10.1186/s13036-023-00401-4.

## Background

Bone defects commonly occur in a variety of orthopaedic diseases. Large bone defects can lead to physical disability and economic burden, harming both individuals and society [1, 2]. Therefore, the treatment of bone defects is particularly important. Over the past few decades, bone tissue engineering has offered promising strategies for repairing bone defects, including the implantation of three-dimensional (3D) porous scaffolds at the defect site to guide and stimulate new bone formation. Conventional approaches to preparing porous polymeric bone repair scaffolds include phase separation [3], particulate leaching [4], and foaming [5]. The above preparation approaches have many disadvantages, mainly that the microstructure and size are difficult to control, the micropores are not connected, and the macro shape cannot be customized. In recent years, 3D printing technology has attracted the attention of many researchers because it can be used to easily prepare micropores suitable for cell growth and scaffolds with a customized macro shape [6–8]. Classic 3D-printed scaffolds are mostly made of synthetic polymeric materials, including polycaprolactone (PCL), poly(lactic acid-glycolic acid) (PLGA), and polylactide (PLA) [7, 9, 10]. These polymeric materials have the advantages of good biocompatibility, biodegradability and good mechanical properties. However, the disadvantage of these synthetic polymers is the lack of osteogenic potential to induce bone regeneration [11]. To address these shortcomings and achieve improved material properties, many researchers have added calcium phosphate (TCP), calcium sulfate, hydroxyapatite (HA), bioactive glass (BG) and other inorganic materials to the scaffold due to the chemical similarity of inorganic materials to natural bone minerals [12–14]. Among various inorganic materials, BG has attracted much attention in the field of bone repair. BG is an inorganic material composed of CaO, SiO_2_ and P_2_O_5_ that has good biocompatibility, degradability, mechanical properties and osteogenic ability [15]. In contrast to HA, TCP, and calcium sulfate, the degradation of BG releases Si, which is an essential trace element in the body [16]. A lack of Si in the body can lead to bone deformation and cartilage tissue defects [17]. Si is involved in early-stage bone formation through regulating the synthesis of collagen [18]. When BG contacts body fluids, HA layers can form, creating a stronger interface between the materials and bone tissue [19]. Furthermore, BG can improve the rigidity and hydrophilicity of polymeric scaffolds. Therefore, 3D-printed scaffolds composed of high-molecular-weight polymers and BG are considered excellent artificial bone graft materials due to their high osteogenic ability, suitable degradation rate and biosafety.

Although 3D-printed polymer/BG scaffolds have many advantages, their surface properties differ from those of the protocellular ECM. Unmodified 3D-printed scaffolds often lack an intrinsic dynamic microenvironment and the necessary physical and chemical properties to guide cell behaviour. It would be appealing to endow these scaffolds with an ECM-mimicking architecture and properties to promote bone regeneration. Interestingly, the emergence of functional hydrogels provides many possibilities to endow 3D-printed scaffolds with hydrophilic networks analogous to those of the ECM. Hydrogels are widely used in the repair of bone, epidermis, nerve and other tissues due to their 3D network structure and high water content, which are conducive to nutrient exchange and metabolite excretion [20, 21]. Furthermore, hydrogels can be used as drug or cell carriers to fill irregular tissue defects [22]. However, hydrogels lack sufficient mechanical and structural integrity when implanted into sites requiring support, so there are certain limitations when applied to repair large bone defects. Integrating 3D-printed scaffolds and hydrogels may be a smart strategy to enhance both the resulting mechanical strength and osteogenic ability compared to those of traditional hydrogels and 3D-printed scaffold materials. Hernandez et al. [23] prepared a novel composite scaffold by coating a 3D-printed PCL scaffold with a self-assembled peptide hydrogel, which showed high hydrophilicity, appropriate biological activity and significant osteogenic differentiation ability. More importantly, hydrogels have good processability and can be designed to adjust the key factors modulating bone regeneration, including growth factor delivery, mechanical stimulation, and cell-to-cell communication [24, 25]. Therefore, the integration of 3D-printed scaffolds and hydrogels offers a promising method for developing biomaterials for bone repair.

Bone repair scaffolds should also provide a suitable microenvironment to recruit stem cells to defect sites and subsequently stimulate the osteogenic differentiation of stem cells; however, 3D-printed scaffolds and hydrogels generally lack this capability. Another traditional way to promote bone regeneration in the treatment of bone defects is the topical use of bioactive factors and drugs to reverse the tissue growth-inhibiting environment and accelerate bone tissue healing [26, 27]. Another feature of hydrogels must be mentioned here: hydrogels can serve as effective drug carriers due to the cross-linking hydrogel network, which can encapsulate and release bioactive molecules [28]. Therefore, combining 3D-printed scaffolds/hydrogels with bioactive factors is a useful approach to enhance the osteogenic and angiogenic activity of scaffolds for bone repair. Stromal cell-derived factor-1α (SDF-1α), also known as CXCL12, is a member of the CXC chemokine family and can specifically bind to CXC chemokine receptor 4 (CXCR4) cell membrane receptors, providing key signals for cell mobilization and homing to specific organs or tissues [29]. Many studies have shown that the SDF-1α/CXCR4 axis plays an important role in promoting bone marrow mesenchymal stem cell (BMSC) recruitment to bone defects through chemotaxis [30]. Furthermore, SDF-1α can promote cell proliferation and activate osteoblasts, leading to satisfactory bone regeneration. SDF-1α can also amplify signal transduction through synergistic action with other regulatory factors [31]. Niu et al. [32] loaded SDF-1α into a silicified collagen scaffold and conducted a series of in vitro and in vivo experiments, and the results showed that the silicified collagen scaffold could slowly release SDF-1α in vitro and that the released SDF-1α could induce the migration of mesenchymal stem cells (MSCs) and vascular endothelial progenitor cells. In addition to the above advantages, SDF-1α promotes angiogenesis and can thus help provide a sufficient blood supply to new tissues and contribute to a better regenerative effect. Therefore, this tissue engineering technique of loading SDF-1α into scaffold materials could serve as a new approach to promote bone tissue repair and regeneration by inducing endogenous stem cells to migrate to damaged tissues.

**Scheme 1:**
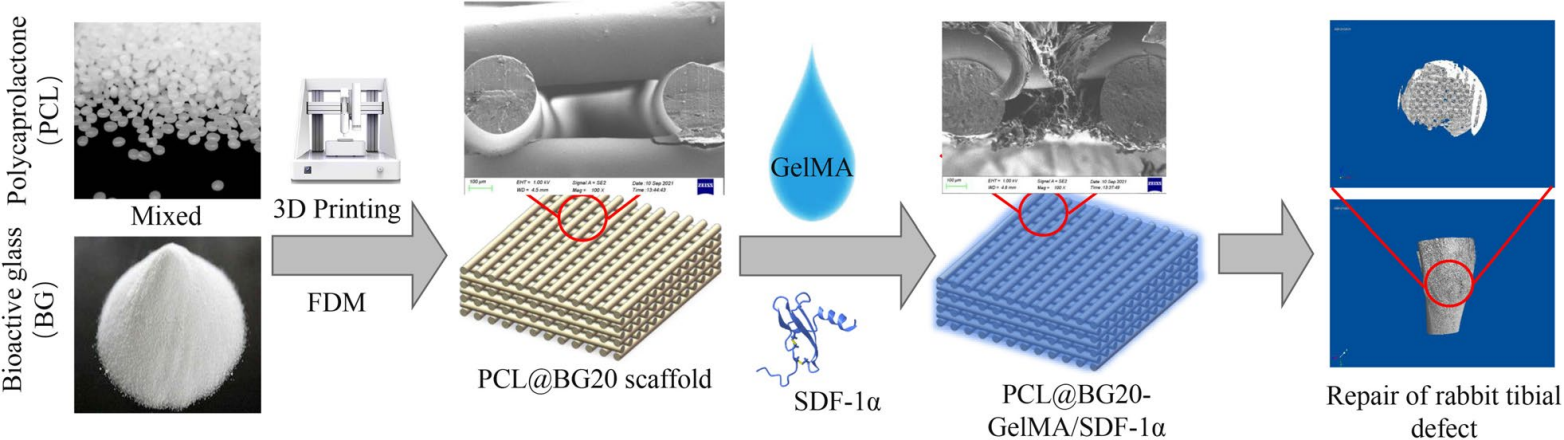
Diagram illustration of the 3D-printed PCL@BG20GelMA scaffold loaded with GelMA hydrogel and SDF-1α for bone regeneration

Herein, we engineered a GelMA hydrogel loaded with SDF-1α and integrated it with 3D-printed PCL@BG20 to develop a bioactive scaffold material for enhanced bone regeneration (Scheme 1). We found that the synthesized scaffold (called PCL@BG20-GelMA/SDF-1α) not only had desirable mechanical properties and hydrophilicity but also enhanced the ability of rat BMSCs (rBMSCs) to migrate. At 8 weeks after implantation in vivo, the PCL@BG20-GelMA/SDF-1α scaffold had significantly accelerated new bone formation, and the new bone tissue showed a higher bone mineral density and larger total bone volume than that in the other groups. Hence, we envision that this hydrogel-3D-printed scaffold construct has great potential for promoting bone tissue regeneration.

## Methods

### Materials

PCL (CAS: 24980-41-4, M_n_=80,000) was purchased from Aladdin Reagents Co., Ltd., China. BG (CAS: 65997-18-4, Mw = 343.37) was purchased from Kunshan Overseas Chinese Technology New Material Co., Ltd., China. GelMA with a degree of substitution of 55-65% was purchased from Beijing SunP Biotech Co., Ltd., China. All chemical reagents were purchased from Beijing Chemical Reagent Co., Ltd. A crystal violet staining kit, DAPI and phosphate buffer solution (PBS) were purchased from Beijing Solarbio Biotechnology Co., Ltd., China. Haematoxylin and eosin (H&E) and Masson staining kits were purchased from Wuhan Servicebio Co., Ltd., China.

### rBMSC culture and identification

As previously mentioned, rBMSCs were isolated from the femoral bone marrow tissues of SD rats aged 3 to 4 weeks [33]. Then, rBMSCs were cultured in a cell culture dish in a 5% CO_2_ humidified incubator at 37 °C. The medium was replaced every two days, the rBMSCs were passaged every 5 days, and cells between passages 2 and 5 were used for experiments. Subsequently, CXCR4 fluorescence staining was used to identify the rBMSCs. Briefly, the cells were fixed with 4% paraformaldehyde, and then 10% goat serum was added to seal the cells for 30 min. Then, AF488-labelled antibodies against cell surface chemokine receptors were added, and the cells were incubated for 1.5 h at 37 °C. After the cells were washed with PBS three times, they were incubated with DAPI for 3 min to label cell nuclei. Finally, the rBMSCs were observed by fluorescence microscopy (Nikon TE-2000U, Japan).

### Preparation and characterization of SDF-1α

The SDF-1α gene was synthesized by a DNA synthesizer and then inserted into the PET21b expression plasmid (Shenzhen BGI Co., Ltd.). The plasmid PET21b-SDF-1α was then transformed into BL21. The strain containing PET21b-SDF-1α was induced to express SDF-1α by 0.2 mM IPTG at 25 °C. Purified SDF-1α was obtained by Ni-NTA affinity chromatography and G25 column desalting. SDF-1α was analysed by 15% SDS‒PAGE electrophoresis. Western blot (WB) analysis of SDF-1α was performed using rabbit anti-human SDF-1α primary antibody (Proteintech, China) and HRP-labelled goat anti-rabbit IgG secondary antibody (Proteintech, China).

### Transwell cell migration assay and quantitative analysis

rBMSCs migration was evaluated using an 8-µm pore-size Transwell system. Briefly, rBMSCs were dissociated into single cells and resuspended in culture medium at a density of 2.5 × 10^5^ cells/mL. Then, 0 µM, 0.5 µM, 1 µM or 2 µM SDF-1α was added to the bottom chamber in the same medium. Subsequently, 100 µL of cell suspension was added to the top cavity of the Transwell, and the cells were cultured for 24 h. After 24 h, the medium in the well was removed, the cells were rinsed 2–3 times with PBS, and the cells were then fixed with 4% paraformaldehyde for 30 min. Finally, the cells were stained with crystal violet solution for 25 min, and then the cells were observed and imaged with a microscope. For quantitative analysis of cell migration, crystal violet-stained samples were treated with 1 mL of 33% acetic acid solution for 1 h to desorb the crystal violet, and the absorbance of the solution at 595 nm was read using a multifunction microplate scanner (Tecan Infinite M200, Switzerland).

### Preparation and characterization of the PCL@BG20-GelMA/SDF-1α scaffold

PCL@BG20 composite particles were prepared by mixing 20 g of BG and 80 g of PCL in an internal mixer (Yiyang Rubber and Plastics Machinery Group Co., Ltd.) at 40 rpm and 100 °C for 10 min. The 10 × 10 × 3 mm scaffold model file was designed using SolidWorks 2017 software. PCL@BG scaffolds were printed from PCL@BG20 particles using a biological 3D printer (3DCreator 02, Ubbiotech, China). The nozzle size of the print head was 0.4 mm, the print head temperature was 100 °C, the filling rate was 50%, the hotbed temperature was 25 °C, and the printing speed was 8 mm/s. These scaffolds were scanned using microcomputed tomography (Micro-CT) (Scansky1172, Bruker), and the porosity was calculated. Hydrogel was then prepared by mixing 2 µM SDF-1 and 20% w/v GelMA in PBS under rotary mixing at 37 °C for 1 h. Then, 3D-printed PCL@BG20 scaffolds were distributed in 24-well plates and immersed in 1 mL of prepared GelMA hydrogel. Subsequently, a 405-nm light source was used to crosslink the GelMA hydrogel for 1 min. After preparation, the scaffolds were frozen at -20 °C for 2 h and then lyophilized for 12 h.

Each scaffold was cut into 5 × 5 × 3 mm pieces, and these small pieces of scaffold were sprayed with gold. The surface and cross-sectional morphology of the scaffold was observed and analysed by SEM (Gemini 2, Zeiss, Germany) at 1 kV. The GelMA/SDF-1α@PCL/BG scaffold was immersed in 2 ml of PBS solution and incubated at 150 rpm at 37 °C for 15 days. Every 2 days, the concentration of SDF-1α in the supernatant was measured using an ELISA kit (Proteintech, China). The tensile strength and compressive strength of the scaffold were measured using a universal mechanical tester (5500, Instron, USA). The 3D-printed scaffolds used in the tensile and compression tests were 10 × 40 × 4 mm and 10 × 10 × 10 mm, respectively. Three parallel samples were tested for each group. Energy dispersive X-ray spectroscopy (EDS Oxford, UK) was used to scan the scaffold surface for elemental N and Si. Fourier transform infrared spectroscopy (FTIR) (Bruker INVENIO-R, Germany) and thermogravimetric analysis (TGA) (TA TGA55, USA) were used to characterize the chemical composition of the scaffolds in the different groups.

### Repair of rabbit proximal tibial defects

Female New Zealand white rabbits were randomly divided into four groups (PCL, PCL@BG20, PCL@BG20-GelMA, and PCL@BG20-GelMA/SDF-1α groups) and used to test the bone repair performance of the scaffolds. All experimental animals were provided by Liaoning Changsheng Biotechnology Company. New Zealand white rabbits were anaesthetized by an intramuscular injection of xylazine hydrochloride at a dose of 0.2 ml/kg; then, the hind leg hair of each rabbit was removed, and the skin was disinfected with iodine. Subsequently, the skin of the experimental animals was cut open, the muscle was bluntly separated, and the proximal tibia was exposed. As shown in Fig. S1, a full-thickness bone defect with a diameter of 7 mm was constructed using a sterile drill in the lower platform of the rabbit tibia. Then, the bone fragments were removed, and the drilled hole was rinsed. The scaffold was implanted into the defect site, and the epidermal wound was sutured. Within 7 days after surgery, penicillin was injected intramuscularly into all experimental animals at a daily dose of 400,000 units. The diet, lower limb activity, defecation and wound healing of New Zealand white rabbits were observed every day.

### Radiographical evaluation

At postoperative week 8, all the experimental New Zealand white rabbits were sacrificed by air injection through the auricular vein. Then, the soft tissue of the lower extremities was removed, and the tibia of the experimental animals was soaked in 4% paraformaldehyde. The effects of the different scaffolds on bone defect healing were examined with high-resolution micro-CT (Bruker SkyScan 1172, Germany). Scanning was performed with a resolution of 15 μm, voltage of 45 kV, and current of 145 µA. After the scan, 3D reconstruction of the bone tissue was performed using CTAn software. Statistical analysis of the bone tissue volume/total tissue volume (BV/TV) in each group was performed.

### Histological evaluation

At postoperative week 8, bone tissue samples were removed and fixed with paraformaldehyde. Then, bone samples were completely decalcified in 15% EDTA solution. After decalcification, bone tissue samples were embedded in paraffin wax and cut into tissue sections. The tissue sections were stained with Masson trichrome, H&E and sirius red in accordance with standard protocols, and samples were observed using a light microscope and polarization microscope.

### Biosafety assessment

At 8 weeks after the operation, the experimental animals were sacrificed, and one healthy New Zealand white rabbit from each group was selected as the control. The heart, liver, spleen, lungs and kidneys were removed from all animals. All visceral tissue was fixed with paraformaldehyde and cut into tissue sections. Subsequently, tissue sections were stained with H&E in accordance with standard protocols, and the samples were observed using a light microscope to evaluate the biological safety of the scaffold.

### Statistical analysis

Statistical analysis was performed using one-way analysis of variance to determine significant differences. Data are expressed as the mean ± SD. A *P* value less than 0.05 was considered to indicate statistical significance in all analyses.

## Results and discussion

### Characterization of SDF-1ɑ and its role in rBMSC recruitment

**Fig. 1:**
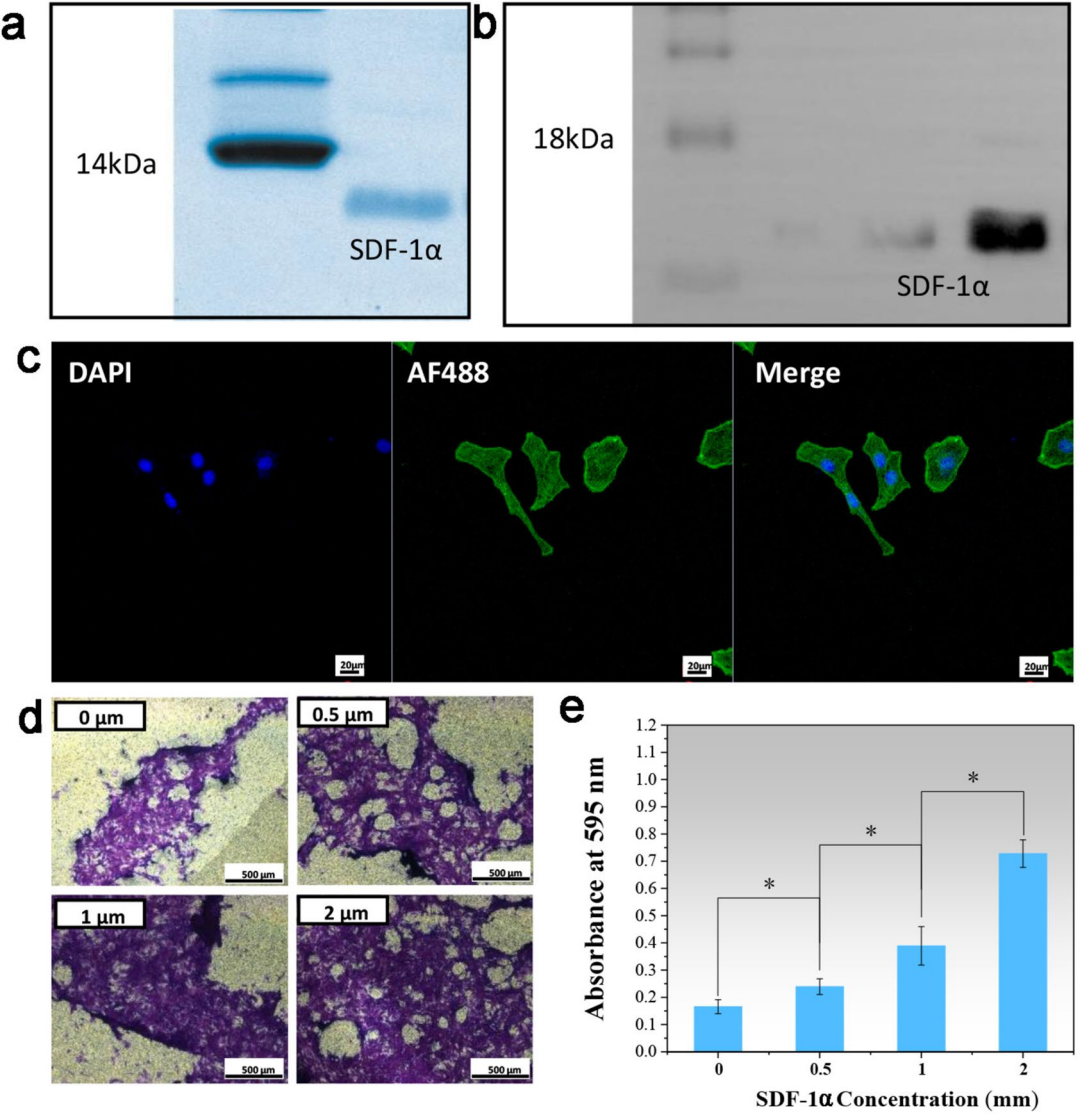
**a** SDF-1α SDS‒PAGE electrophoretic diagram. **b** WB analysis of SDF-1α at different concentrations. **c** AF488-labelled CXCR4 immunofluorescence identification of rBMSCs; scale bars represent 40 μm. **d** Transwell cell migration assay. The cells were stained with crystal violet; the scale bars represent 500 μm. **e** The corresponding quantitative evaluation of cell migration in different concentrations of SDF-1α, *P* < 0.05; error bars represent standard deviation for *n* = 3

As shown in Fig. 1a, pure SDF-1α protein was obtained by Ni-NTA affinity chromatography and desalting chromatography, and the molecular weight was consistent with the theoretical molecular weight of 9.0 kDa. As shown in Fig. 1b, the WB results showed a single band at 9.0 kDa, which further indicated that the purified protein was SDF-1α. The SDF-1α/CXC signalling pathway plays an important role in stem cell migration and is the main reason that SDF-1α can recruit stem cells. Therefore, we identified the presence of the CXCR4 receptor on the surface of our extracted rBMSCs by immunofluorescence staining. As shown in Fig. 1c, the immunofluorescence results showed that there were a large amount of CXCR4 protein on the surface of rBMSCs; thus, CXCR4, as a target on the cell surface, could be recruited by SDF-1α. Cell migration is the slow directional movement of cells through cell body deformation after receiving endogenous or exogenous signals. Many studies have found that SDF-1α has a chemotactic effect on BMSCs [34]. Therefore, we determined the concentration of SDF-1α with the strongest chemotactic effect by measuring the number of cells on the Transwell membrane. The Transwell chemotaxis assay results showed that SDF-1α promoted rBMSC migration in a dose-dependent manner (Fig. 1d and e) compared to the control. SDF-1α significantly promoted rBMSC migration starting at 0.5 µmol. The in vitro data showed that with increasing SDF-1α concentration, the ability of the chemokine SDF-1α to promote rBMSC migration gradually improved. According to the experimental results, a concentration of 2 µmol/mL SDF-1α was used in the subsequent experiments.

### Characterization of the GelMA/SDF-1α@PCL/BG scaffold

**Fig. 2:**
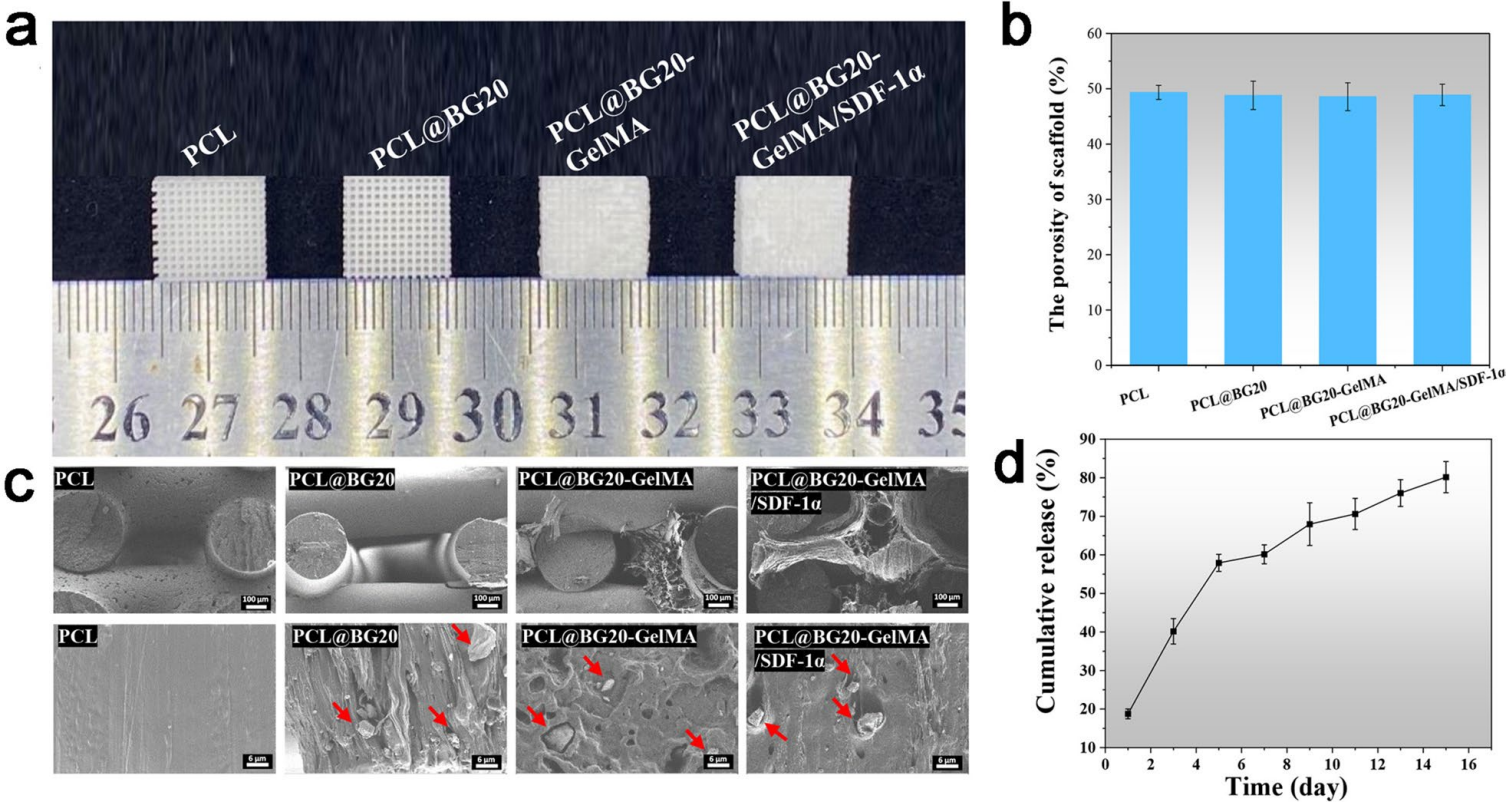
**a** Macroscopic view and (**b**) porosity of the scaffold. **c** SEM images of different scaffolds. The red arrows indicate BG particles. **d** SDF-1α cumulative release curve from the GelMA/SDF-1α@PCL/BG scaffold, *n* = 3

The appearance of the scaffold was white, and the scaffold is presented in Fig. 2a as a 10 × 10 × 3 mm cube. The PCL and PCL@BG20 scaffolds showed an obvious porous structure. The porous structures of PCL@BG20-GelMA and PCL@BG20-GelMA/SDF-1α were covered by GelMA. As shown in Fig. 2b, the porosity of the PCL scaffold was 49.34%, and that of the scaffolds containing 20% BG ranged between 48.57% and 48.88%. As shown in Fig. 2c, SEM revealed the surface and cross-section of the PCL scaffold to be relatively smooth, with no GelMA between the pores. The cross-sectional SEM images showed BG particles uniformly dispersed in PCL@BG20, PCL@BG20-GelMA and PCL@BG20-GelMA/SDF-1α. After GelMA and GelMA/SDF-1α hydrogels were added to the scaffold, hydrogels with a highly porous structure formed between the scaffold pores. As shown in Fig. 2d, SDF-1α was released continuously during GelMA hydrogel degradation for up to 15 days, indicating a sustained cytokine delivery function of PCL@BG20-GelMA/SDF-1α. In our previous research, we explored the effects of different proportions (5–20%) of BG in PCL on osteogenic differentiation, and the results showed that PCL scaffolds containing 20% BG had the strongest ability to promote osteogenesis; thus, 20% BG was used in this study [35].

**Fig. 3:**
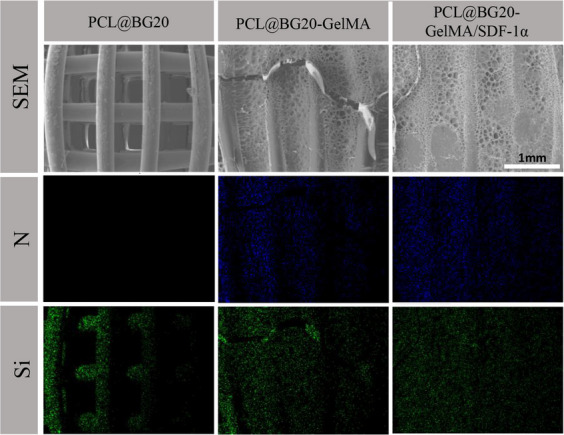
Contents of Si and N in different scaffolds analysed by EDX mapping. The scale bar represents 1 mm

**Fig. 4:**
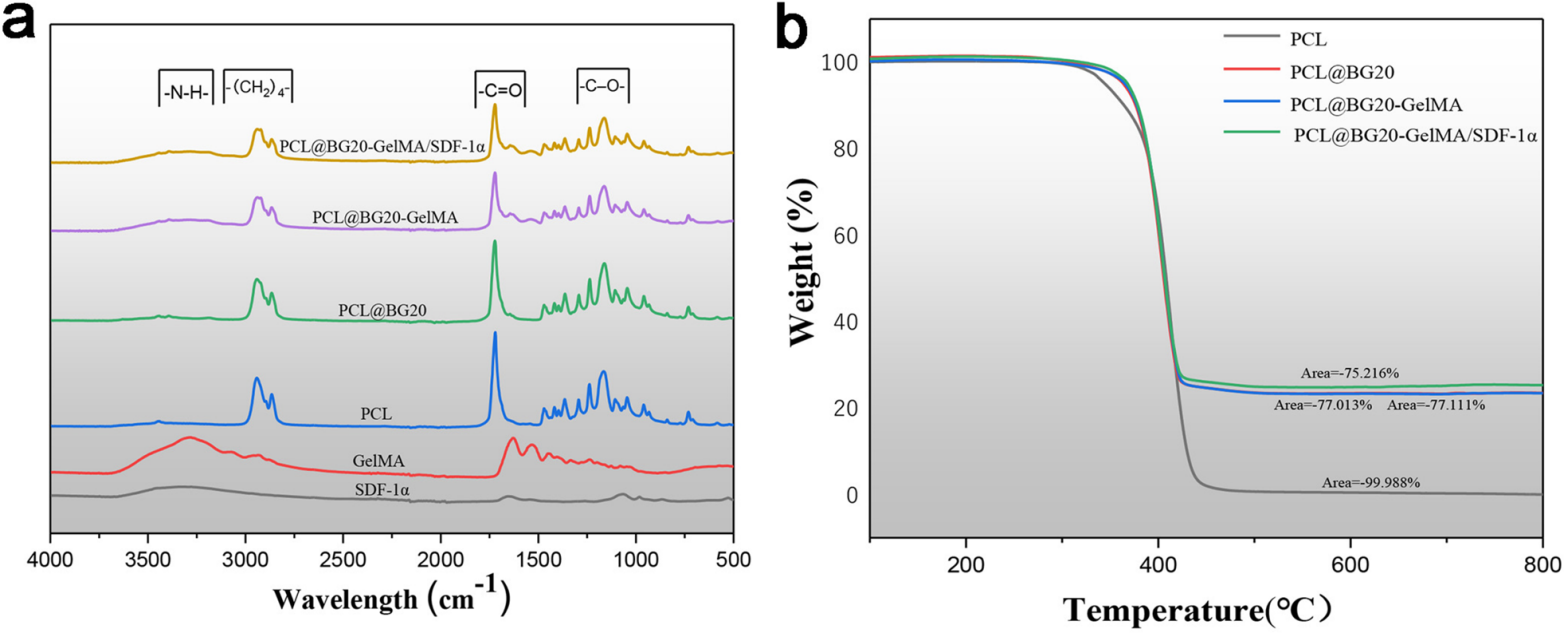
**a** FTIR spectra and (**b**) TGA curves of different scaffold materials

The results of the surface element analyses of different scaffolds are shown in Fig. 3. The EDS maps showed Si (blue) exposure on the surface of the PCL@BG, PCL@BG20-GelMA and GelMA/SDF-1α@PCL/BG scaffolds. Compared with the PCL/BG scaffold, new elements (N) could be seen on the surface of the PCL@BG20-GelMA/SDF-1α scaffold, indicating successful immobilization of the hydrogel on both scaffolds. Subsequently, FTIR was used to further study the chemical composition of the different scaffold materials. As shown in Fig. 4a, FTIR revealed the characteristic peaks of PCL at 2850–3000 cm^−1^ (representing - (CH_2_)_4_ -), 1750 cm^−1^ (representing -C = O), and 1150–1250 cm^−1^ (representing -(- C-O-)). Compared with the PCL scaffold, the PCL@BG20 scaffold showed no visible alteration in the FTIR spectrum, indicating that the BG was dispersed into the PCL only by physical mixing instead of by chemical reaction. The FTIR spectrum of GelMA showed a newly formed vibration peak of the amide bond at 1680 cm^−1^, indicating that the methacrylate group had been successfully grafted onto the gelatine skeleton. On the other hand, a new band at approximately 3400 cm^−1^, which represented the stretching vibration of the alcoholic hydroxyl group, appeared on the PCL@BG20-GelMA and PCL@BG20-GelMA/SDF-1α scaffolds. Furthermore, another band at 1550–1660 cm^−1^ was observed in the spectra of PCL@BG20-GelMA@ and PCL@BG20-GelMA/SDF-1α, which represented –NH_2_. There were no –NH_2_ groups in the PCL or PCL@BG20 scaffolds. We also tested the mass ratios of various components in the scaffolds using TGA. As shown in Fig. 4b, with increasing temperature, PCL, GelMA and SDF-1α in the scaffold decomposed successively, and the remaining substance in the scaffold was BG. The TGA results showed that the mass percentage of BG in the PCL@BG20, PCL@BG20-GelMA and PCL@BG20-GelMA/SDF-1α scaffolds was 22.987%, 22.889% and 24.784%, respectively.

**Fig. 5:**
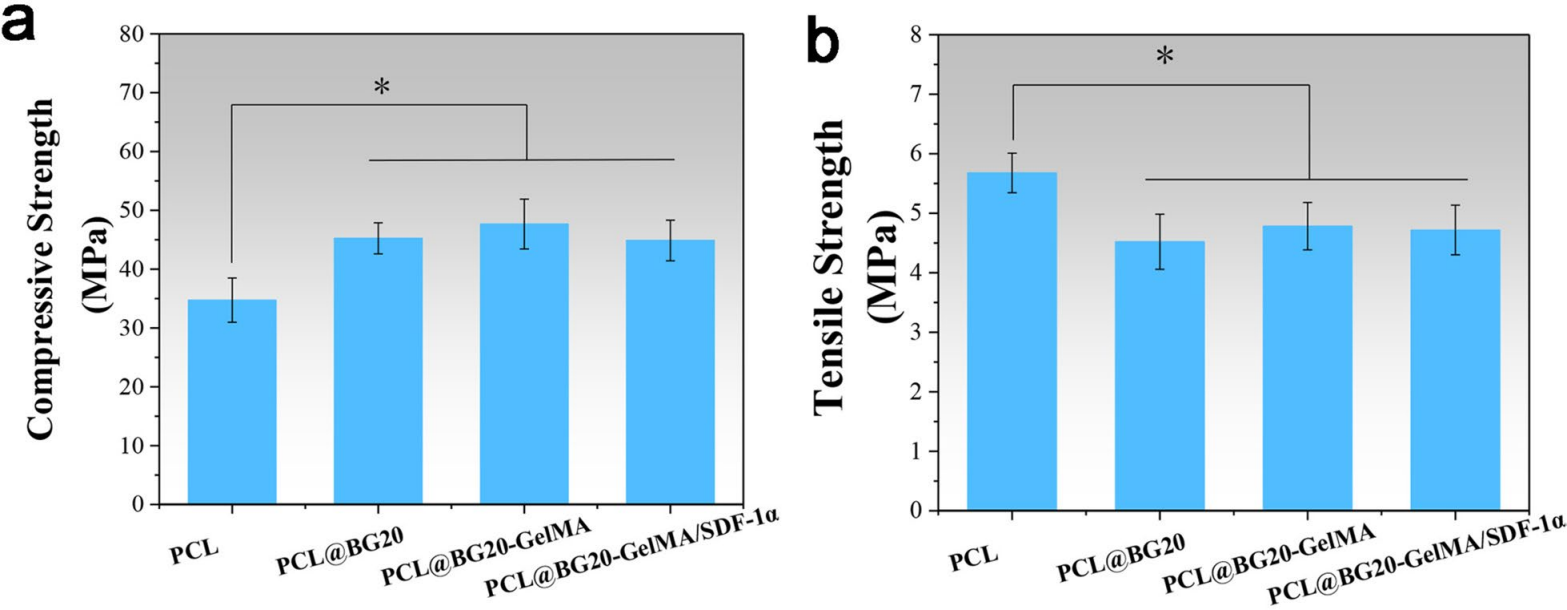
**a** compression strength and (**b**) tensile strength of different scaffolds. *P < 0.05, error bars represent standard deviation for *n* = 3

In the treatment of bone defects, scaffolds must not only fill the bone defect site but also provide mechanical support. Therefore, the mechanical strength of scaffolds is very important for bone repair, especially at load-bearing sites. In this study, the compressive and tensile strengths of different scaffold materials were analysed by mechanical tests. As shown in Fig. 5a and b, the compressive and tensile strength of the pure PCL scaffold was 34.73 ± 3.77 MPa and 5.68 ± 0.33 MPa, respectively. After the addition of BG, the compressive strength of the scaffolds increased to 45.24 ± 2.63 MPa, but the tensile strength of the scaffolds decreased to 4.52 ± 0.46 MPa, which indicates that BG can effectively improve the stiffness of the materials but decreases their flexibility. After the addition of GelMA and SDF-1α, the compressive and tensile strength of the scaffold did not change significantly, so the addition of GelMA and SDF-1α had little effect on the mechanical properties of the scaffold. Chen et al. [36] found that the compression modulus of GelMA was somewhat related to the degree of methacryloyl substitution and that GelMA with a degree of substitution below 65% had little effect on the compression modulus of scaffolds. According to the above results, the compressive strength of the PCL@BG20-GelMA/SDF-1α scaffold was determined to be similar to that of human cancellous bone, making this scaffold an ideal bone replacement material.

### In vivo bone repair efficacy of the PCL@BG20-GelMA/SDF-1α scaffold

**Fig. 6:**
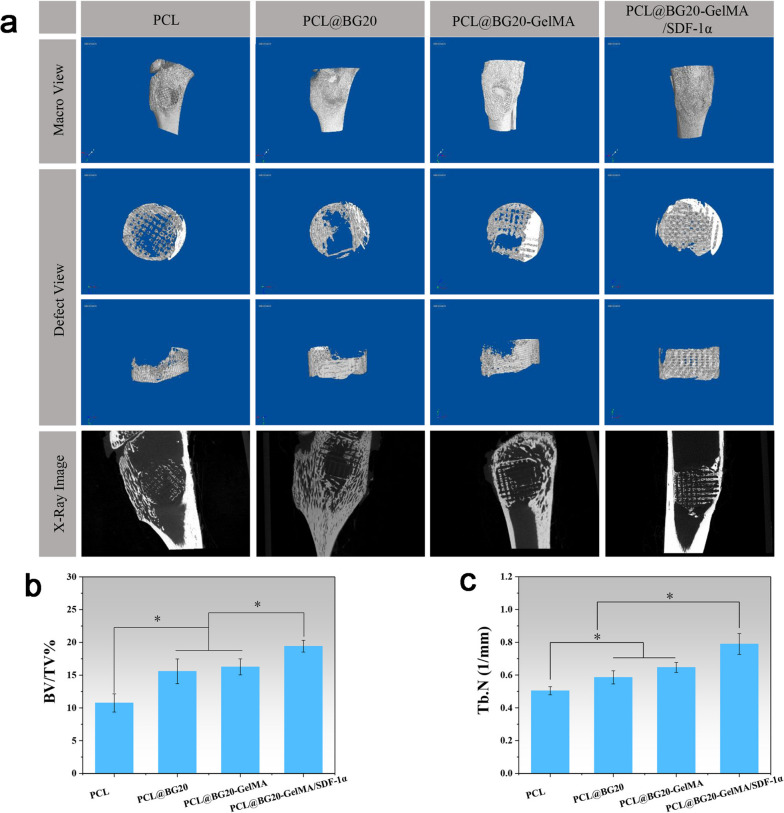
**a** Representative 3D micro-CT images of the region of the proximal radius defect at 8 weeks postsurgery. Quantitative analysis of (**b**) BV/TV and (**c**) Tb.N in new trabecular bone at 8 weeks after surgery. *P < 0.05; error bars represent the standard deviation for *n* = 3

To assess bone formation in different groups, micro-CT images were taken at 8 weeks after surgery (Fig. 6a). Compared with the PCL group, the other groups showed more obvious new bone formation at the defect site, and the density of new bone was also significantly increased. Among all experimental groups, the PCL@BG20-GelMA/SDF-1α group showed the largest area of newly formed bone and large amounts of bone tissue ingrowth into the pores of the scaffold. Sagittal images of the bone tissue also showed that in the PCL@BG20-GelMA/SDF-1α groups, more new bone had formed around the scaffold at the bone defect site. We performed a quantitative analysis of the new bone tissue by 3D reconstruction (Fig. 6b). The BV/TV in the PCL@BG20 group was 15.59 ± 1.88%, which was significantly higher than that in the PCL group (10.76 ± 1.39%). There was no significant difference in the BV/TV between the PCL@BG20 group and the PCL@BG20-GelMA group. The BV/TV was significantly higher in the PCL@BG20-GelMA/SDF-1α scaffold group than in the other groups. As shown in Fig. 6c, the Tb.N in the PCL@BG20 group was obviously higher than that in the PCL group. There was no significant difference in the Tb.N between the PCL@BG20 group and the PCL@BG20-GelMA group. PCL@BG20-GelMA/SDF-1α showed a higher Tb.N than the other groups. As shown in Fig. S2, macroscopic images of each rabbit tibia 8 weeks after implantation of the different scaffolds showed that the bone surface was smooth, the scaffold was completely within the defect, and there was no obvious inflammatory reaction. The above results prove that the combination of BG + GelMA + SDF-1α can significantly improve the biological activity of scaffold materials and has a very good effect on bone tissue regeneration after injury.

### Histological analysis

**Fig. 7:**
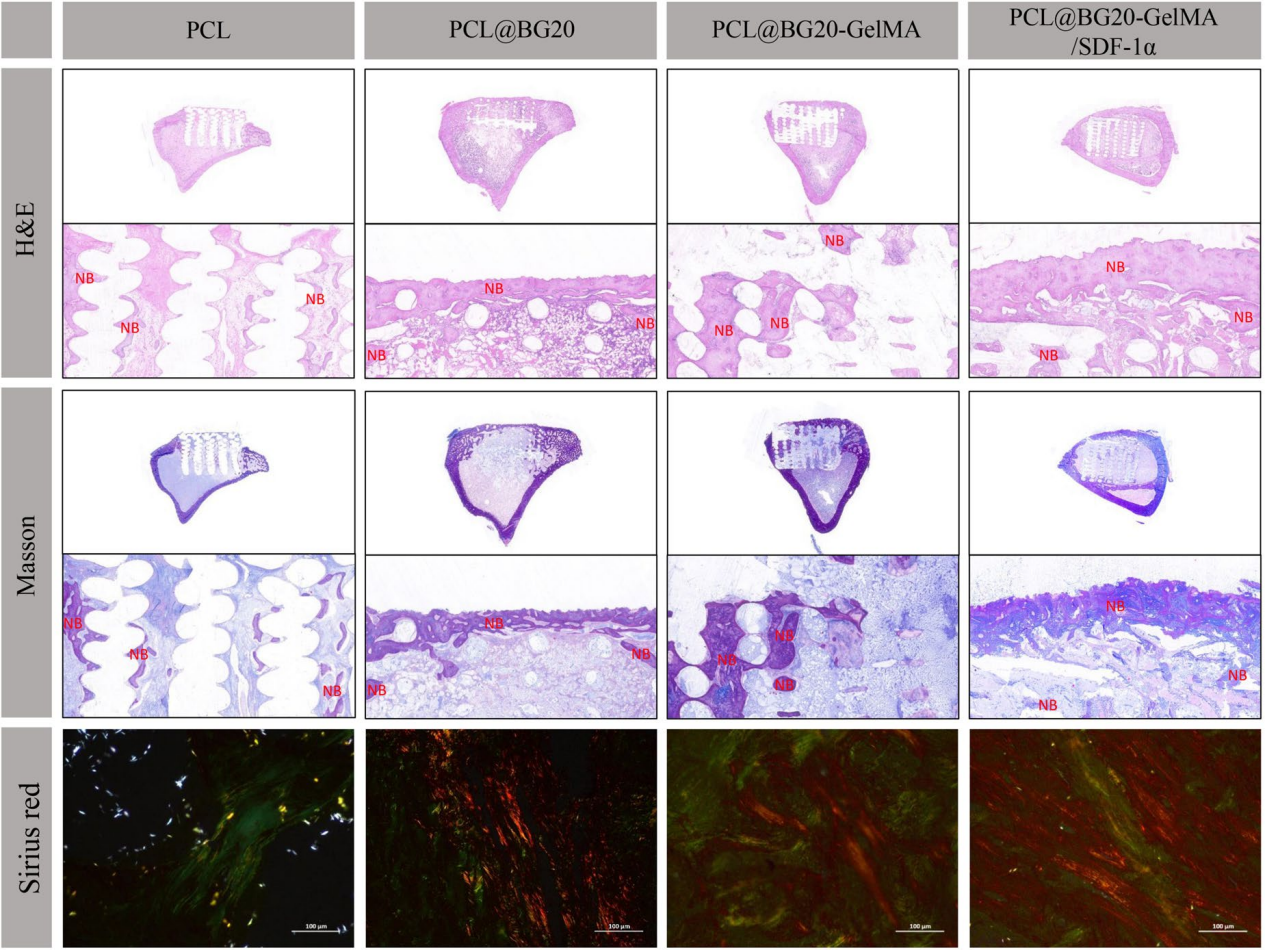
Histological analysis using H&E, Masson trichrome and sirius red staining

Eight weeks after surgery, the rabbits were sacrificed, and bone tissue was removed. Subsequently, we analysed bone formation and collagen deposition at the defect site by H&E, Masson, and sirius red staining. As shown in Fig. 7, the scaffolds in each group were located at the bone defect site, and the scaffolds were fixed well, providing a good framework for the growth of bone tissue. In the PCL group, H&E and Masson staining showed only a small amount of new bone formation in the pores of the scaffold. However, there was a significant increase in the volume and thickness of new bone in the PCL@BG20 and PCL@BG-GelMA groups. Among all the groups, the PCL@BG20-GelMA/SDF-1α group showed the largest amount and greatest thickness of new bone, which was consistent with the results of micro-CT analysis. These results indicate that the addition of SDF-1α can promote the aggregation of rBMSCs, ensure that bone defect sites have a sufficient cell supply, and promote the rapid formation of new bone tissue, which was also consistent with the findings of Wang et al. [37]. Wang et al. [37] found that scaffolds carrying SDF-1α exhibited better bone conduction and induction ability. Scaffold materials can be used to repair large bone defects in a shorter time when carrying SDF-1α, with a repair effect similar to that of autogenous bone. To observe the distribution of collagen fibres in the new bone tissue, the sections were stained with sirius red. As shown in Fig. 7 (sirius red), the collagen fibre area in the PCL@BG20, PCL@BG20-GelMA and PCL@BG20-GelMA/SDF-1α groups was larger than that in the PCL group. Among all groups, type I collagen fibres were best distributed in the PCL@BG20-GelMA/SDF-1α group and arranged in an orderly manner, which indicates good bone tissue formation. However, there were fewer and more disordered collagen fibres in the other groups, indicating inferior bone formation compared to that in the PCL@BG20-GelMA/SDF-1α group and suggesting that the addition of SDF-1α further promoted bone tissue healing. These results further indicate that the recruitment of rBMSCs is important for bone regeneration.

### Scaffold biosafety assessment

**Fig. 8:**
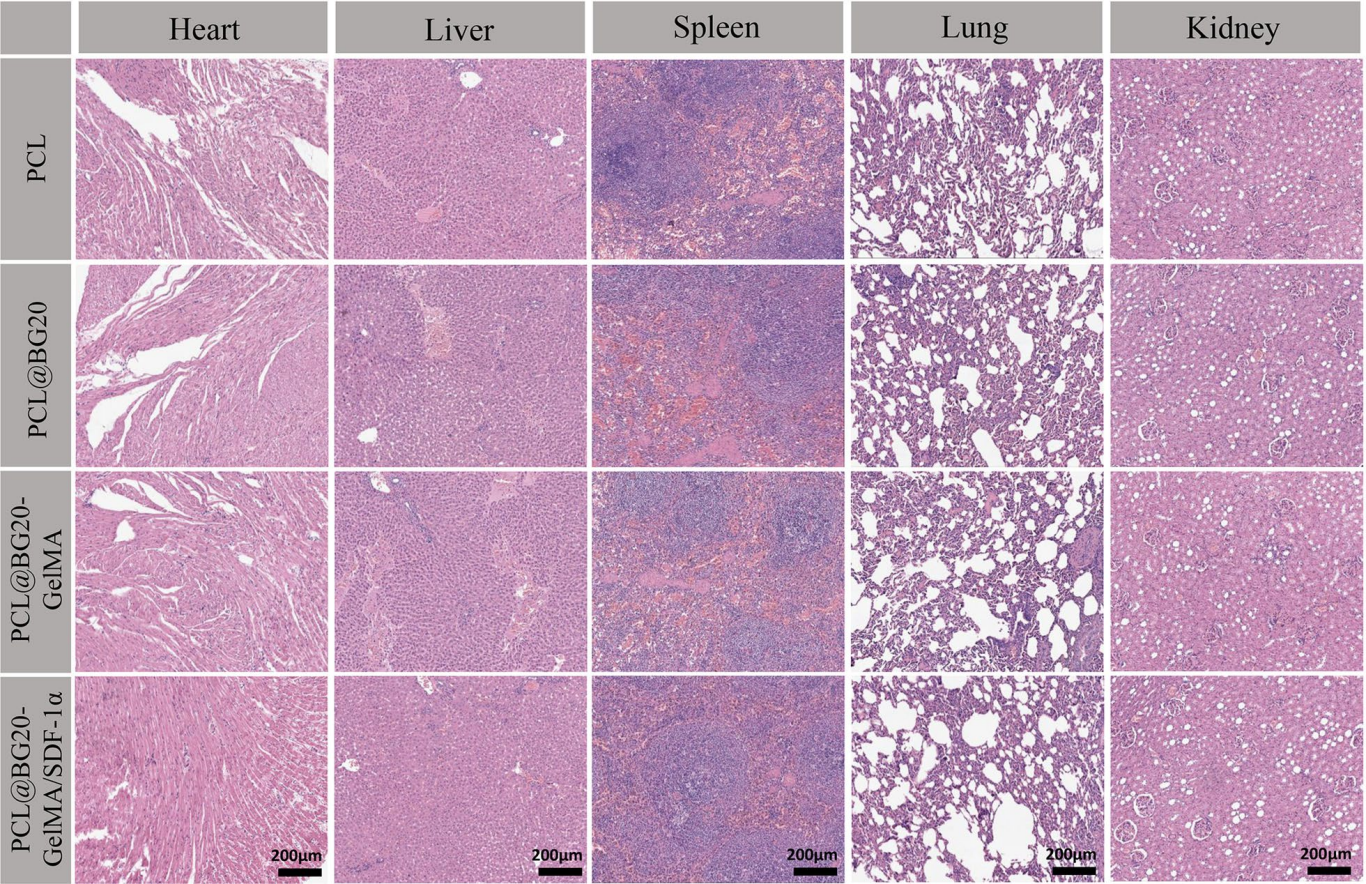
At 8 weeks after surgery, H&E-stained sections of important organs (heart, liver, spleen, lungs and kidneys) were obtained from experimental animals. Scale bars represent 200 μm

Bone tissue imaging and histological experiments confirmed that BG, GelMA and SDF-1α can significantly improve the bone repair ability of PCL scaffolds. However, such nanomaterials can enter other parts of the body through the circulation of body fluids. Therefore, the biosafety of scaffold materials also needs to be studied. The heart, liver, spleen, kidneys and lungs of the experimental animals were observed histopathologically 8 weeks after the operation. As shown in Fig. 8, the results of H&E staining of the heart tissue of healthy rabbits showed that the myocardial fibre tissue arrangement was normal, with no obvious changes to the myocardial interstitium. The structure of the liver lobule was intact, and there was no degeneration or sclerosis of the liver cells. Furthermore, no obvious pathological changes or inflammatory cell infiltration were observed in the spleen, kidneys or lungs. At 8 weeks, no significant differences were found in the heart, liver, spleen, lungs or kidneys of the experimental animals in each group compared with those of healthy rabbits. Therefore, when the PCL@BG20-GelMA/SDF-1α scaffold is applied locally to a bone defect site, it will not have any adverse effects on the important organs of rabbits. The above experimental results indicate the excellent biocompatibility and bone repair ability of the PCL@BG20-GelMA/SDF-1α scaffold we prepared.

3D scaffolds play an important role in the treatment of bone defects by providing suitable mechanical support and architectural clues for bone regeneration. In recent years, PCL scaffolds prepared by 3D printing technology have attracted widespread attention due to their excellent mechanical properties, biocompatibility, and porous structure, which promote bone growth [7]. However, the hydrophobicity and lack of necessary osteogenic activity limit the further application of PCL [38]. In addition to suitable mechanical strength and porosity, materials applied in bone defect repair also need to provide a good regenerative microenvironment to promote cell proliferation, cell differentiation and extracellular matrix synthesis. Therefore, another type of material that is widely used in bone repair is hydrogels. Hydrogel materials exhibit good biocompatibility and encapsulation, allowing them to simulate the structure of the natural extracellular matrix and promote cell growth and nutrient exchange [39]. However, hydrogels also have the disadvantage of low mechanical properties, which are not conducive to their application in load-bearing areas. In this study, the versatility of 3D printing technology enabled us to combine the mechanically strong polymer PCL with the hydrogel GelMA in one structure, fully combining the advantages of both polymeric and hydrogel scaffolds. SEM observation showed that the prepared scaffolds had an appropriate pore size and could effectively promote the diffusion of nutrients and oxygen. Pore structures of 300 μm or larger have been reported to favour cell migration, bone formation, and angiogenesis. Furthermore, the scaffold we prepared had good mechanical strength, similar to that of natural cancellous bone. Thus, the hydrogel could effectively improve the hydrophilicity of the scaffold without negatively affecting its mechanical properties.

Although the combination of hydrogel and 3D-printed PCL scaffolds can effectively overcome the shortcomings of the original materials, the bone induction ability of the materials itself is still insufficient. To further improve the bone repair ability of scaffolds, some bioactive molecules should also be added. In this study, BG and SDF-1α were added to PCL and GelMA hydrogel, respectively, to jointly improve the osteogenic activity of the scaffold. BG is an important bioceramic material with a strong ability to bond with bone. BG can provide temporary support for tissue regeneration during bone healing. Furthermore, BG can ameliorate the hydrophobic properties and poor cell adhesion of polymeric materials and improve their mechanical properties [40]. During degradation, BG releases Ca^2+^, PO4^3−^ and Si^4+^, which contribute to bone regeneration. Our experimental results showed that the mechanical properties of the scaffold material were effectively improved after BG was added. Animal experiments further demonstrated that the addition of BG improved the effect of bone repair.

When tissue-engineered scaffolds are used to treat bone defects, the lack of stem cells in the repair site is often caused by poor integration of the grafts and host tissues. To solve this problem, in this study, SDF-1α was loaded into the scaffold material. SDF-1α is a very important chemokine of stem cells that can bind with CXCR4 on the surface of stem cells and then guide stem cells to migrate to a defect area [29]. Some studies have also found that SDF-1α can protect newborn cells from hypoxic damage, reducing the apoptosis rate and promoting the formation of new blood vessels, thereby creating a more favourable microenvironment for bone tissue regeneration [41, 42]. Our results show that SDF-1α can effectively recruit stem cells. Furthermore, when SDF-1α and BG were used together to modify the scaffold material, the scaffold showed the best effect on bone repair. The PCL@BG20-GelMA/SDF-1α group showed the largest area of new bone and large amounts of bone tissue ingrowth into the pores of the scaffold; additionally, collagen deposition at the defect site was also effectively improved. Furthermore, the scaffold showed high biocompatibility and no obvious toxicity to or side effects on the important organs (liver, spleen, kidneys, lungs and heart) of rabbits. Therefore, we believe that the PCL@BG20-GelMA/SDF-1α scaffold we prepared has good clinical development prospects and may play an essential role in bone tissue regeneration medicine.

## Conclusions

In summary, we prepared a new bone repair scaffold material by combining a 3D-printed PCL@BG20 scaffold with GelMA/SDF-1α hydrogel, which has a suitable biomimetic structure, excellent mechanical properties, hydrophilicity and ability to induce osteogenesis. The addition of SDF-1α into the PCL@BG20-GelMA scaffold endowed the scaffold with a strong ability to recruit stem cells. The compressive strength of the PCL@BG20-GelMA/SDF-1α scaffold was 44.89 ± 3.45 MPa, which is similar to that of natural cancellous bone. The combined application of SDF-1α and BG significantly improved the mechanical properties and osteogenic activity of the scaffold. The results of in vivo experiments showed that the PCL@BG20-GelMA/SDF-1α scaffold had a good effect on the repair of proximal tibial defects in rabbits. Successful regeneration of bone tissue was confirmed by mineralized collagen deposition and increased bone content. This study shows that the simultaneous use of SDF-1α and BG for the functional modification of bone implants is a promising strategy and that PCL@BG20-GelMA/SDF-1α scaffolds should serve as excellent bone grafts for bone defect repair.

### Supplementary Information

**Additional file 1: Fig.S1.** The images of before and after scaffold implantation. **Fig.S2.** Macroscopic images of each rabbit tibia 8 weeks after implantation of the different scaffolds.

## Data Availability

Not applicable.

## Abbreviations

PCL: Polycaprolactone
BG: Bioactive glass
BMSCs: Bone marrow stromal cells
3D: Three-dimensional
PLGA: Poly(lactic acid-glycolic acid)
PLA: Polylactide
GP: Graphene
HA: Hydroxyapatite
ECM: Extracellular matrix
SDF-1α: Stromal cell-derived factor-1α
MSCs: Mesenchymal stem cells
PFA: Paraformaldehyde
PBS: Phosphate buffer solution
HE: Haematoxylin and eosin
EDS: Energy dispersive spectroscopy
Micro-CT: Microcomputed tomography
ROI: Region of interest
CXCR4: CXC chemokine receptor 4
WB: Western blot
FDM: Fused deposition modelling
