# Supplementary material for: 3D-printed PCL@BG scaffold integrated with SDF-1α-loaded hydrogel for enhancing local treatment of bone defects
Source: J Biol Eng. 2024 Jan 2;18:1. doi: 10.1186/s13036-023-00401-4 (PMC10763424; doi:10.1186/s13036-023-00401-4)
Supplement: Supplementary file 1 — Additional file 1: Fig.S1. The images of before and after scaffold implantation. Fig.S2. Macroscopic images of each rabbit tibia 8 weeks after implantation of the different scaffolds. [file 13036_2023_401_MOESM1_ESM.docx]

**Supplementary material**

# 3D-printed PCL@BG scaffold integrated with SDF-1α-loaded hydrogel for enhancing local treatment of bone defects

Chenglong Wang^1,2^, Jinlei Dong^1,2^, Fanxiao Liu^1,2^, Nan Liu^1,2^, Lianxin Li^1,2*^

^1^ Department of Orthopaedics Surgery, Shandong Provincial Hospital Affiliated to Shandong First Medical University, Jinan 250021, China

^2^ Department of Orthopaedics Surgery, Shandong Trauma Center, Jinan 2500021, China

**^*^Corresponding author**

Email: 13505312449@163.com


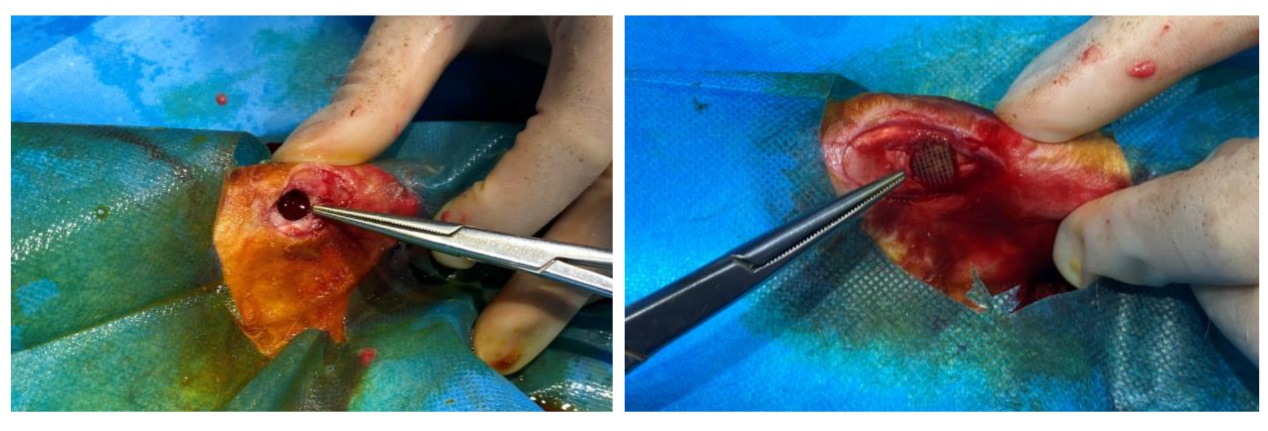


Fig.S1. The images of before and after scaffold implantation.


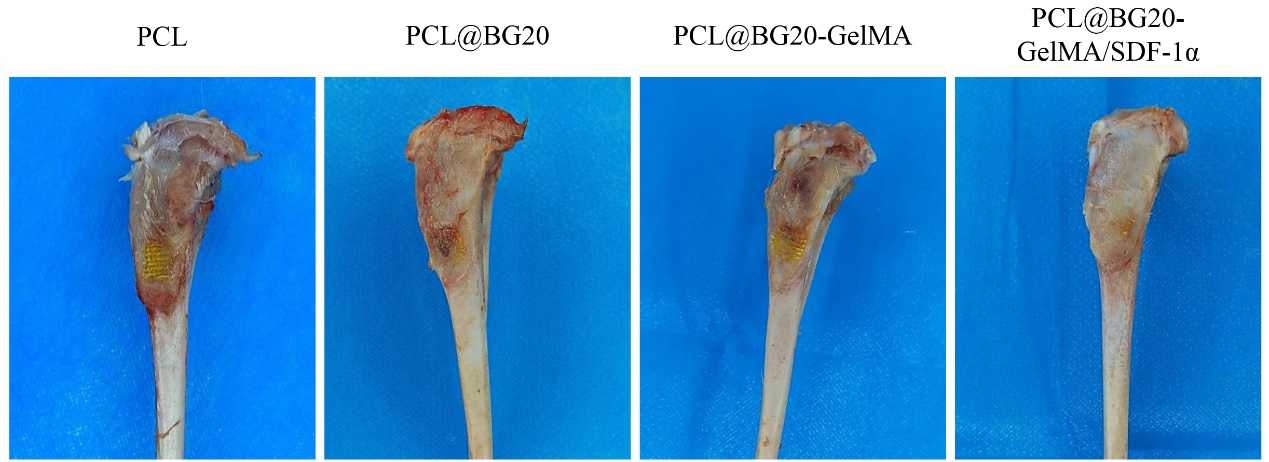


Fig.S2. Macroscopic images of each rabbit tibia 8 weeks after implantation of the different scaffolds.
